# Supplementary material for: Deconvolution of conformational exchange from Raman spectra of aqueous RNA nucleosides
Source: Commun Chem. 2020 May 6;3:56. doi: 10.1038/s42004-020-0298-x (PMC9814580; doi:10.1038/s42004-020-0298-x)
Supplement: Supplementary file 1 — Supplementary Information [file 42004_2020_298_MOESM1_ESM.pdf]

Supplementary Tables and Figures

Deconvolution of Conformational Exchange from Raman Spectra of Aqueous RNA Nucleosides

Supplementary Table 1 | Assignment of characteristic experimental vibrations of each solvated ribonucleoside to specific vibrational modes. The predicted Raman spectrum of the single, most-populous conformer from each classical MD distribution was used.

● – In-plane
○ – Out-of-plane
○*def* - Out-of-plane deformations

ν- Stretching (ν_{sym} – Symmetric, ν_{asym} – Asymmetric)
δ – Scissoring
ω - Wagging
T - Twisting
ρ - Rocking

Experimental characteristic Raman-active vibrational modes / cm^{-1}				Assignment / cm^{-1}			
U	C	A	G	U	C	A	G
	602	606	606		**587** – ●ρ (C-N-C).	**610** – ●ν_{sym} Base and Ribose Ring.	**577** – ●ν_{sym} Ribose Ring.
		613	613		**613** – ●ν_{sym} Base and Ribose Ring.		**617** – ○*def* Base. ω (C-N-C).
629	628			**646** – ●ρ Base and Ribose Ring.	**635** – ●ρ Base and Ribose Ring.		
650				**657** – ●ν C-N in Base.			
		734				**735** – ●ν Base (C-N). ●ν_{sym} Ribose Ring.	
786	786	801	793	**776 –** ○*def* Base (ω N-C-N).	**786 –** ○*def* Base.	**819 –** ●ν Base (C=C). ○ρ Base (O-H).	**796 –** ○ρ / ν_{asym} Base (C=C-C).
873	869	873	881	**866 –** ○*def* Ribose Ring (○ρ C-O-C).	**672 –** ●ν_{sym} Ribose Ring. ○*def* Ribose Ring. (C-N).	**866 –** ○ν_{sym} Ribose Ring (C-C and C-O). **891 –** ●ν_{sym} Ribose Ring (C-C).	**881 –** ●ν_{sym} Base (C=N, C=C). ○*def* Ribose Ring.
916	912			**902 –** ●ν Ribose Ring (C-C) ●ν Base (C-N)	**906 –** ●ν Ribose Ring (C-C) ●ν Base (C-N)		
1002	995	1013		**994 –** ○ν & ω Base (C-C and C-O). ●ν Ribose Ring.	**993 –** ○*def* Base (C=C).	**1001 –** ●ν Base (C=N). ρ (N-H)	

					ρ (C-N and N-H)<br>●ν Ribose Ring (C-O)		
1045	1040			**1048 –**<br>○ρ Ribose Ring (C2'-C3').<br>●ν Base.	**1043 –**<br>○ρ Ribose Ring (C2'-C3').<br>●ν Base.	**1046 –**<br>ν_{asym} (C-O) forcing<br>○*def* Ribose Ring.	
		1059	1062			**1055 –**<br>●ν Ribose Ring (C-C, C-O).<br><br>1068 –<br>●ν and ρ (O-H) Ribose Ring and Base.	**1064 –**<br>●ν_{asym} Ribose Ring (C-C-N).<br>●ν Base (C-N).
1091	1088			**1096 –**<br>●ν (C-O) and ρ (C-H) Ribose Ring.<br>●ν Base.	**1089 –**<br>●ν (C-O) and ρ (C-H) Ribose Ring.<br>●ν_{sym} Base (N-C-N).<br>ρ Base (H-N-H).	**1092 –**<br>●ν Ribose Ring (C-O).<br>ρ (C-H) Ribose Ring.	
1136	1139			**1133 –**<br>○*def/*ρ Ribose Ring (C2'-C3').<br>●ν Base (C-N).<br>ρ Base (C-H).	**1043 –**<br>●ν_{sym} Base (N-C-N and C=C).<br>○ρ Ribose Ring (C2'-C3').		
	1196	1181	1184		**1191 –**<br>○*def* Ribose Ring (C2').<br>ρ Base (C-H).	**1183 –**<br>ω (O-H and C-H).	**1190 –**<br>○*def* Ribose Ring (C2').<br>●ν Base (C-H).
	1218	1217			**1215 –**<br>●ν (C-N) between Ribose Ring and Base<br>ρ (C-N) in Base.<br>●ν Base (C=C, C=N) and ρ (C-H).	**1213 –**<br>●ν Base (C-N).<br>ρ (C-H).<br><br>**1223 –**<br>●ν Base (C=C, C=N)	
1236				**1235 –**<br>○*def* Ribose Ring ($HOCH_2$).<br>ρ Ribose Ring and Base (C-H).			
	1247	1254			**1239 –**<br>ν Between Base and Ribose Ring (C-N).<br>●ν Base (C=C, C-N, C=N).<br><br>**1255 –**<br>○*def* Ribose Ring (HOCH2).	**1256 –**<br>○*def* Ribose Ring (C2').<br>●ν Base	

SUPPLEMENTARY TABLE 1

					●ν (C-C).		
	1297				**1302 –**<br>●ν Ribose Ring (C-C).<br>●ν Base (C-N).<br>ρ (C-H).		
		1311		.		**1312 –**<br>●ν Base (C=N).<br>●ν Ribose Ring.	
			1325				1320 –<br>●ν Base (C-N).<br>ρ Ribose Ring and Base (C-H).
		1341				**1341 –**<br>●ν Base (C-C, C-N and C=N).	**1335 – 1347 –**<br>●ν_{asym} Base (N-C=C and N-C=N).
	1373		1371		**1371 –**<br>ρ Base (C-H and N-H).<br>●ν Base (C=C and C-C).		**1368 –**<br>●ν_{asym} Ribose (C-C-C).
		1382				**1381 –**<br>○ρ/ν Ribose Ring (C-C and $HOCH_2$).	
1400				**1401 –**<br>ν_{asym} Base (C-N-C, C-C).<br>●ν Ribose Ring.<br>ρ Ribose Ring and Base (C-H)			
	1414	1434	1420		**1406 –**<br>●ν Base (C-N).<br>○*def* Ribose Ring (C2’).	**1437 –**<br>●ν Base (C=C and C=N)	**1426 –**<br>●ν (C-N) between Base and Ribose Ring.<br>●ν_{sym} Base (N-C)
	1459	1465			**1436 –**<br>●ν Base.<br>ρ Base and Ribose Ring (C-H).	**1467 –**<br>●ν Base.<br>ρ Base (C-H).	
1478				**1471 –**<br>○*def* ($HOCH_2$)			**1471 –**<br>○*d* ($HOCH_2$)
	1495	1489	1489		**1509 –**<br>●ν Base (C=C. C-N and C=N).<br>ρ (C-H).<br>δ (H-N-H).	**1498 –**<br>●ν Base (C-N and C=N).<br>δ (H-N-H).	**1514 –**<br>●ν_{asym} Base (N-C=N and N-C=C).<br>ρ (C-H).
	1531	1512			**1509 – 1563 –**<br>●ν Base (C=C. C-N and C=N).<br>ρ (C-H).	**1526 –**<br>●ν_{asym} Base (N-C=N).<br>●ν Base (C=C).	

					δ (H-N-H).		
		1588	1582			**1608 –** ●ν_{asym} Base (N-C=N). ●ν Base (C=C). δ (H-N-H).	**1584 –** ●ν_{asym} Base (N-C=N and N-C=C). ●ν Base (C=O). ρ (C-N).
	1612				**1612 –** ●ν Base (C=C, C=O). ●ν_{asym} (C=C-N). δ (H-N-H).		
1631				**1671 –** ●ν Base (C=C). ρ (C-H). ●ν_{sym} (C-N-C).			
		1641	1641			**1644 –** ●ν_{asym} (N=C-C, C=C-C).	**1649 –** ●ν_{asym} (N=C-N). δ (H-C-H). ●ν Base (C=O)
	1654				**1654 –** ν_{asym} Base. δ (H-N-H). ρ (C-H).		
1685				**1671 –** ●ν Base (C=C). ρ (C-H). ●ν_{asym} (C-N-C).			

Supplementary Table 2 | Ribonucleoside conformer energies relative to the *syn*/south conformer (kJ/mol). Geometry optimization of 1000 frames from each classical MD simulation allowed for extraction of the lowest energy conformer from each of the four major conformational regions. For the purines, *syn*/south was the lowest energy conformer, whereas for pyrimidines, *anti*/south was the lowest energy conformer.

	**purine nucleosides**		**pyrimidine nucleosides**	
conformation	*A*	*G*	*C*	*U*
syn/south	+0.0	+0.0	+0.0	+0.0
syn/north	+9.0	+6.2	+2.1	+3.1
anti/south	+5.4	+10.7	-7.7	-4.7
anti/north	+24.3	+19.5	-3.6	+3.2

SUPPLEMENTARY TABLE 3

Supplementary Table 3 | Comparison between the predictions of conformational preferences from 5μs molecular dynamics simulations (with explicit TIP3P water) using a range of force-fields.

	purine nucleosides		pyrimidine nucleosides	
conformation	*A*	*G*	*C*	*U*
syn/south	53%	29%	4%	4%
syn/north	31%	55%	<1%	<1%
anti/south	3%	2%	60%	52%
anti/north	13%	14%	36%	44%

The OPLS-AA/M force field for RNA: Robertson, MJ *et al.* (2019) Development and testing of the OPLS-AA/M force-field for RNA. *J. Chem. Theory Comput.* **15**, 2734-2742.

	purine nucleosides		pyrimidine nucleosides	
conformation	*A*	*G*	*C*	*U*
syn/south	70%	61%	65%	61%
syn/north	27%	35%	14%	22%
anti/south	2%	2%	15%	13%
anti/north	1%	2%	6%	4%

The AMBER ff14SB biomolecular force field. Maier, JA *et al.* (2015). ff14SB: improving the accuracy of protein side chain and backbone parameters from ff99SB. *J. Chem. Theory Comput.* **11**, 3696-3713.

	purine nucleosides		pyrimidine nucleosides	
conformation	*A*	*G*	*C*	*U*
syn/south	32%	26%	17%	17%
syn/north	15%	28%	8%	10%
anti/south	25%	15%	25%	26%
anti/north	28%	31%	50%	47%

The AMBER ROC RNA force field. Aytenfisu, AH *et al.* (2017). Revised RNA dihedral parameters for the amber force field improve RNA molecular dynamics. *J. Chem. Theory Comput.* **13**, 900-915.

	purine nucleosides		pyrimidine nucleosides	
conformation	*A*	*G*	*C*	*U*
syn/south	8%	8%	2%	3%
syn/north	5%	10%	<1%	<1%
anti/south	50%	47%	75%	78%
anti/north	37%	35%	23%	19%

The AMBER GAFF force field for small molecules. Wang, J *et al.* (2004). Development and testing of a general AMBER force field. *J. Comp. Chem.* **25**, 1157-1174.

SUPPLEMENTARY TABLE 4

Supplementary Table 4 | Angular distribution of 9 randomly selected adenosine canonical conformers *pre* and *post* geometry optimization. It is clear that optimization did not result in significant changes to geometries. This observation is consistent across all four nucleosides.

χ		**P**	
Pre	*Post*	*Pre*	*Post*
55.2	59.6	75.1	32.6
171.6	-174.4	347.1	8.4
65.5	65.1	8.1	41.9
55.9	75.1	175.6	154.8
57.6	53.2	28.8	23.2
53.5	57.6	24.8	19.6
62.5	63.9	154.0	160.1
51.9	54.0	159.5	167.3
83.7	75.3	42.9	46.4

SUPPLEMENTARY FIGURE 1

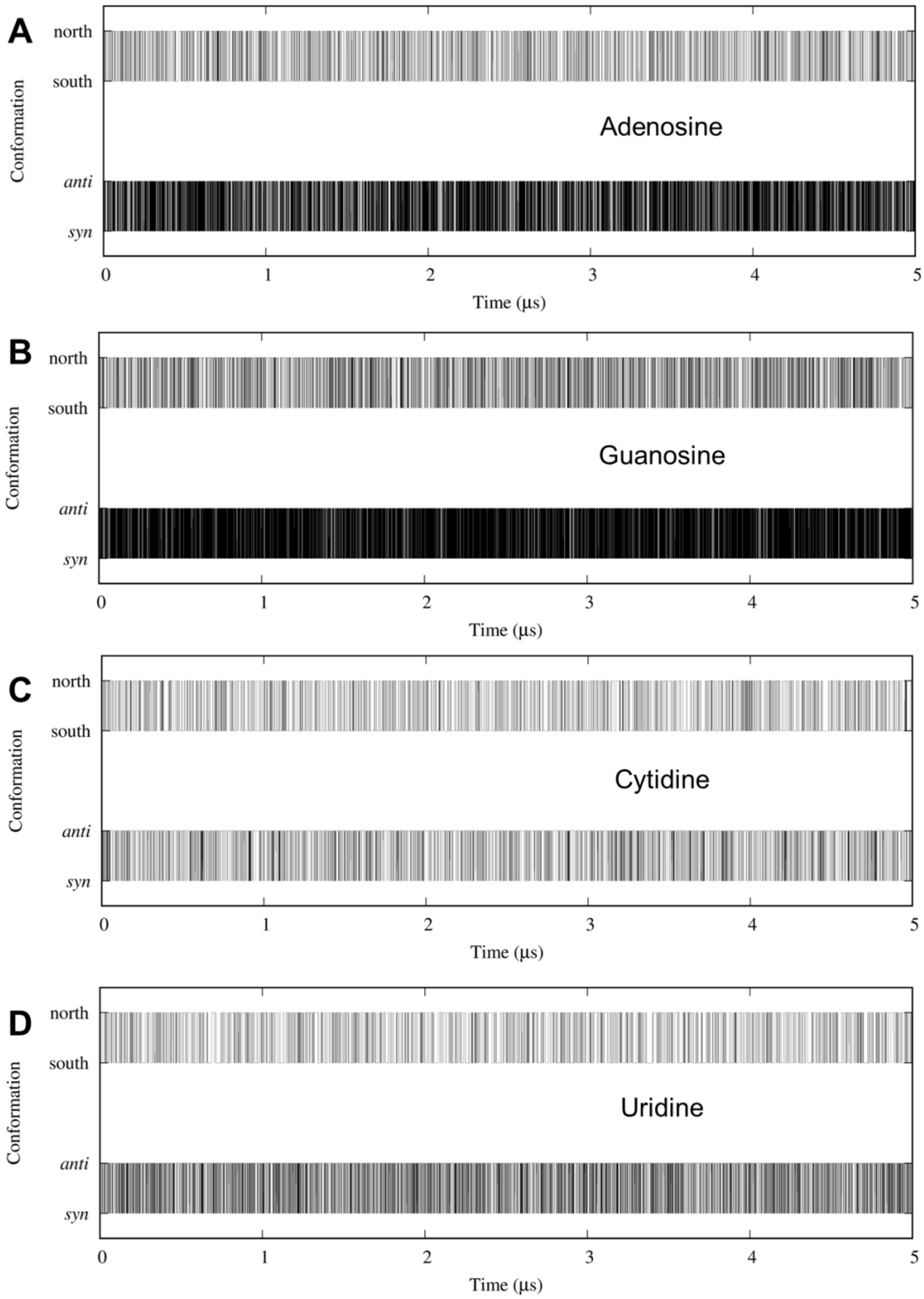

Supplementary Figure 1 | Interconversion between major solvated ribonucleoside conformers throughout each 5 μs classical MD simulation for adenosine (A), guanosine (B), cytidine (C), and uridine (D); each point represents 100 ps. Rapid interconversion between the four major conformers is observed throughout each simulation, with the total populations shown in Fig. 2.

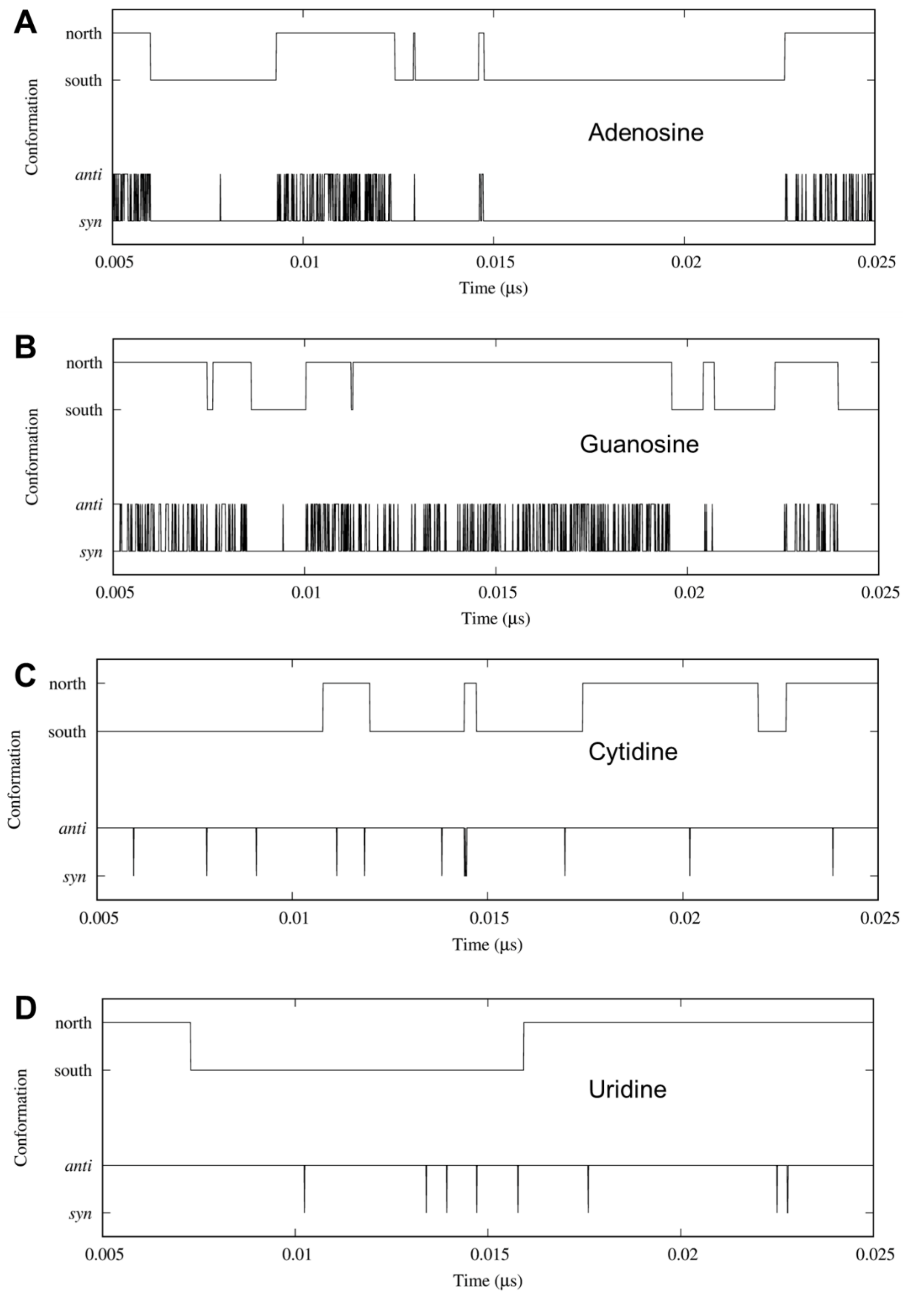

Supplementary Figure 2 | Interconversion between major solvated ribonucleoside conformers throughout a 20 ns window from each 5 µs classical MD simulation for adenosine (A), guanosine (B), cytidine (C), and uridine (D); each point represents 10 ps. Rapid interconversion between the four major conformational states is observed.

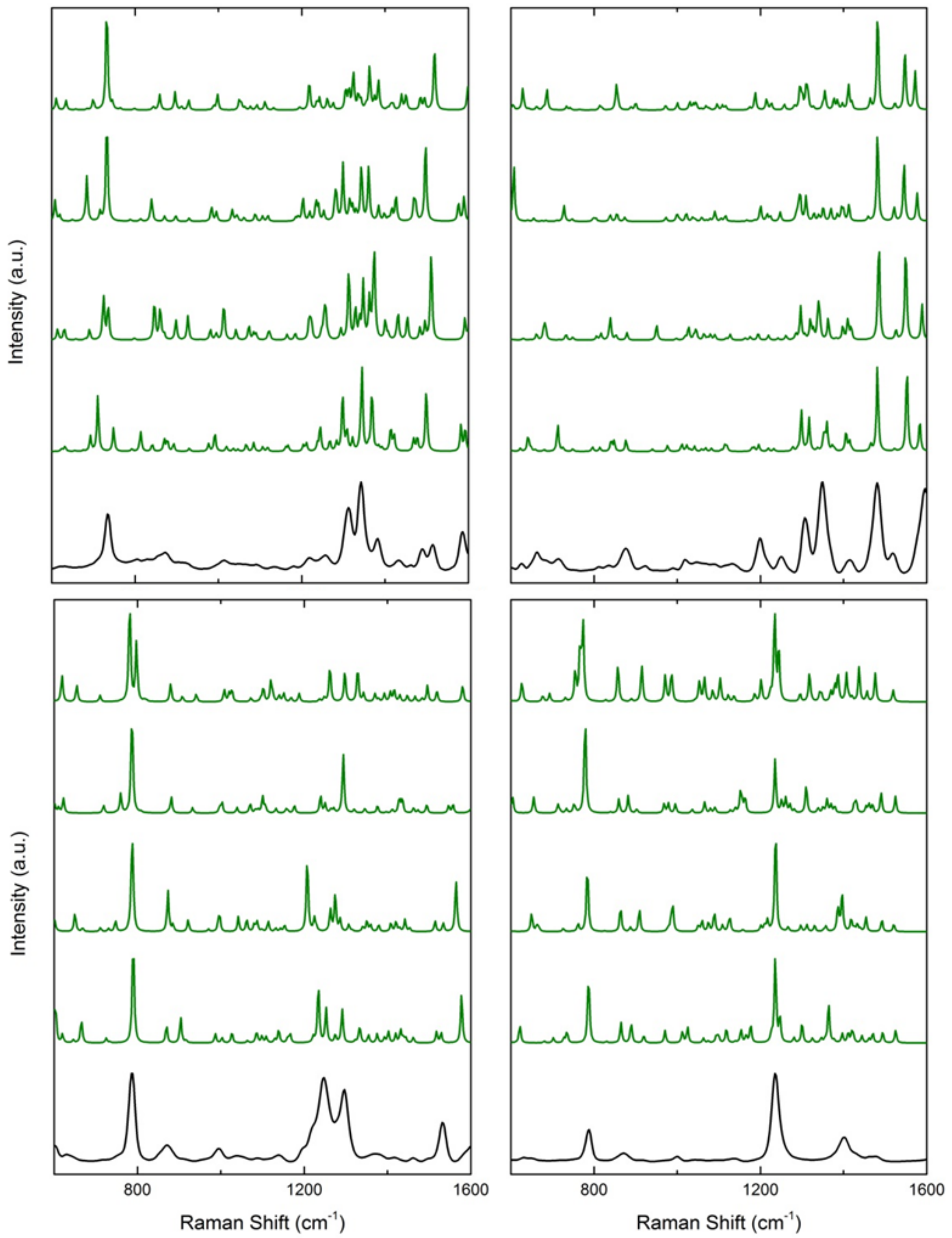

Supplementary Figure 3 | Comparison of DFT calculations in canonical conformations (from top to bottom *syn*/south, *syn*/north, *anti*/south, *anti*/north). Adenosine, top left, guanosine, top right, cytidine, bottom left, and uridine, bottom right.

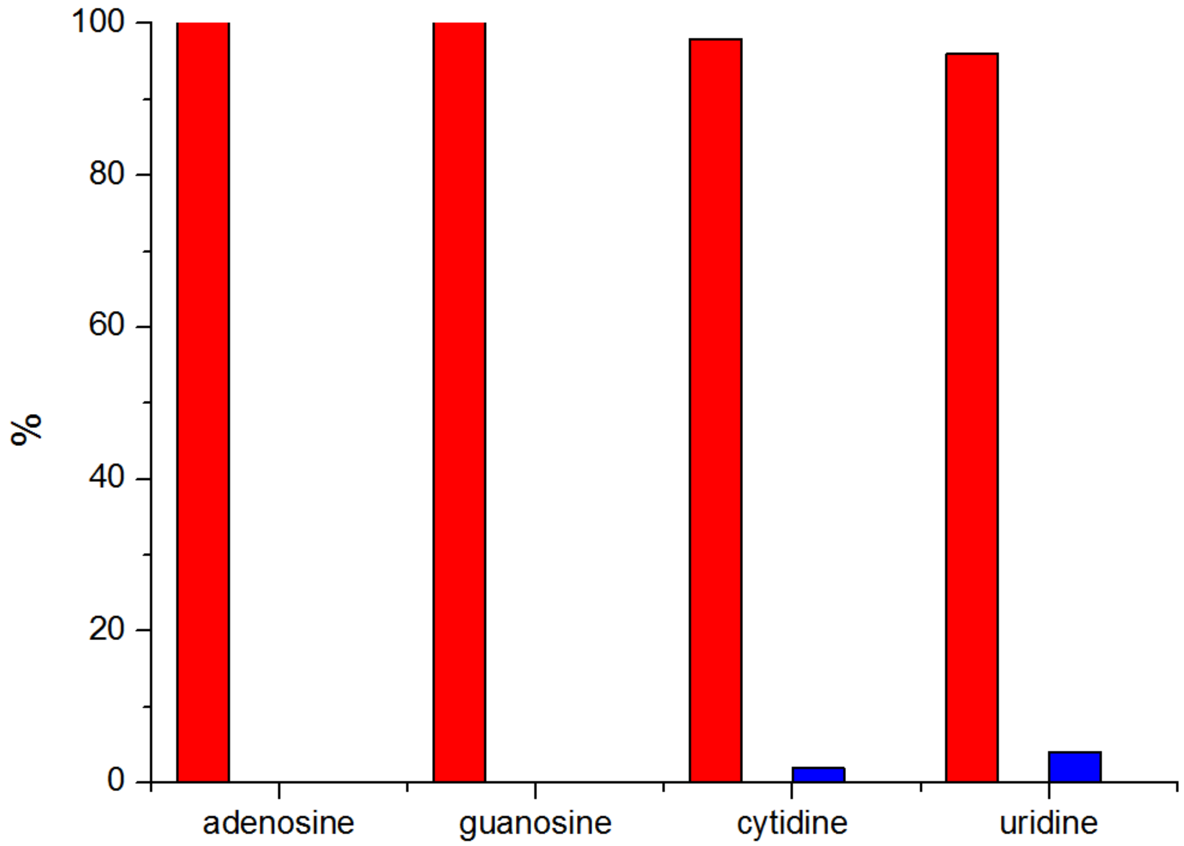

Supplementary Figure 4 | *Ab initio* molecular dynamics simulations of the local dynamics around the *syn*/south (red) conformer of each solvated ribonucleoside. Each AIMD simulation (17.5 ps) was started from a structure with a *syn*/south conformation, and did not stray significantly into the *syn*/north (green), *anti*/south (blue), or *anti*/north (cyan) regions.

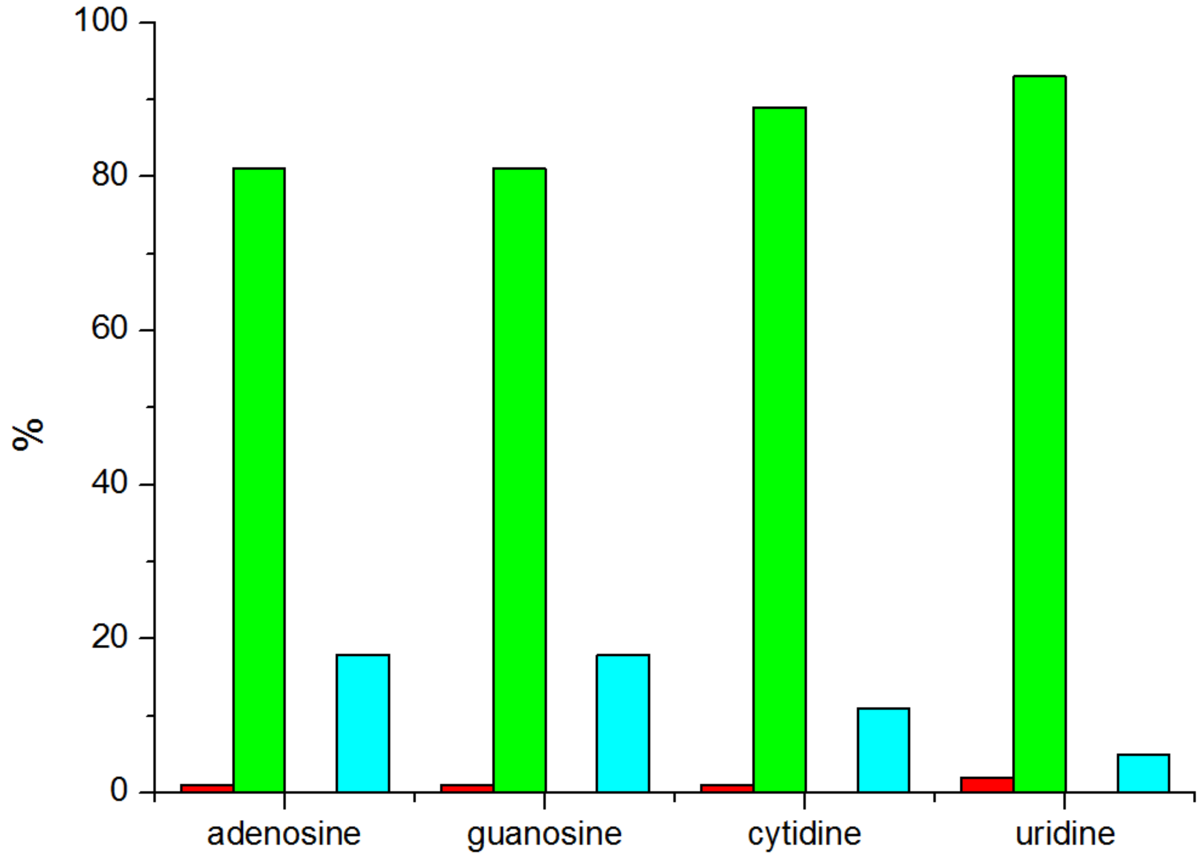

Supplementary Figure 5 | *Ab initio* molecular dynamics simulations of the local dynamics around the *syn*/north (green) conformer of each solvated ribonucleoside. Each AIMD simulation (17.5 ps) was started from a structure with a *syn*/north conformation, and did not stray significantly into the *syn*/south (red), *anti*/south (blue), or *anti*/north (cyan) regions.

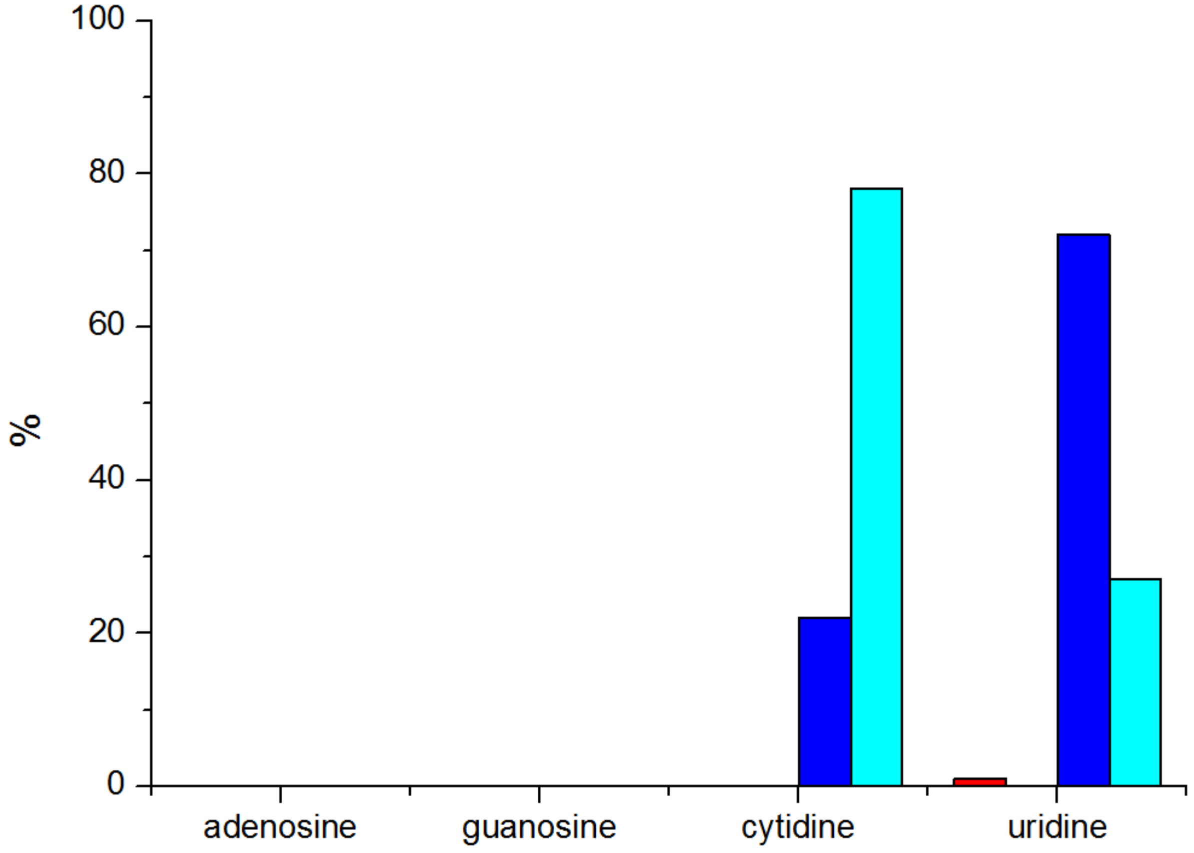

Supplementary Figure 6 | *Ab initio* molecular dynamics simulations of the local dynamics around the *anti*/north-south (cyan/blue) conformers of solvated cytidine and uridine. Each AIMD simulation (17.5 ps) was started from a structure with an *anti*/south conformation, and did not stray significantly into the *syn*/south (red) or *syn*/north (green) regions. However, facile interchange was observed between *anti*/south and *anti*/north throughout.

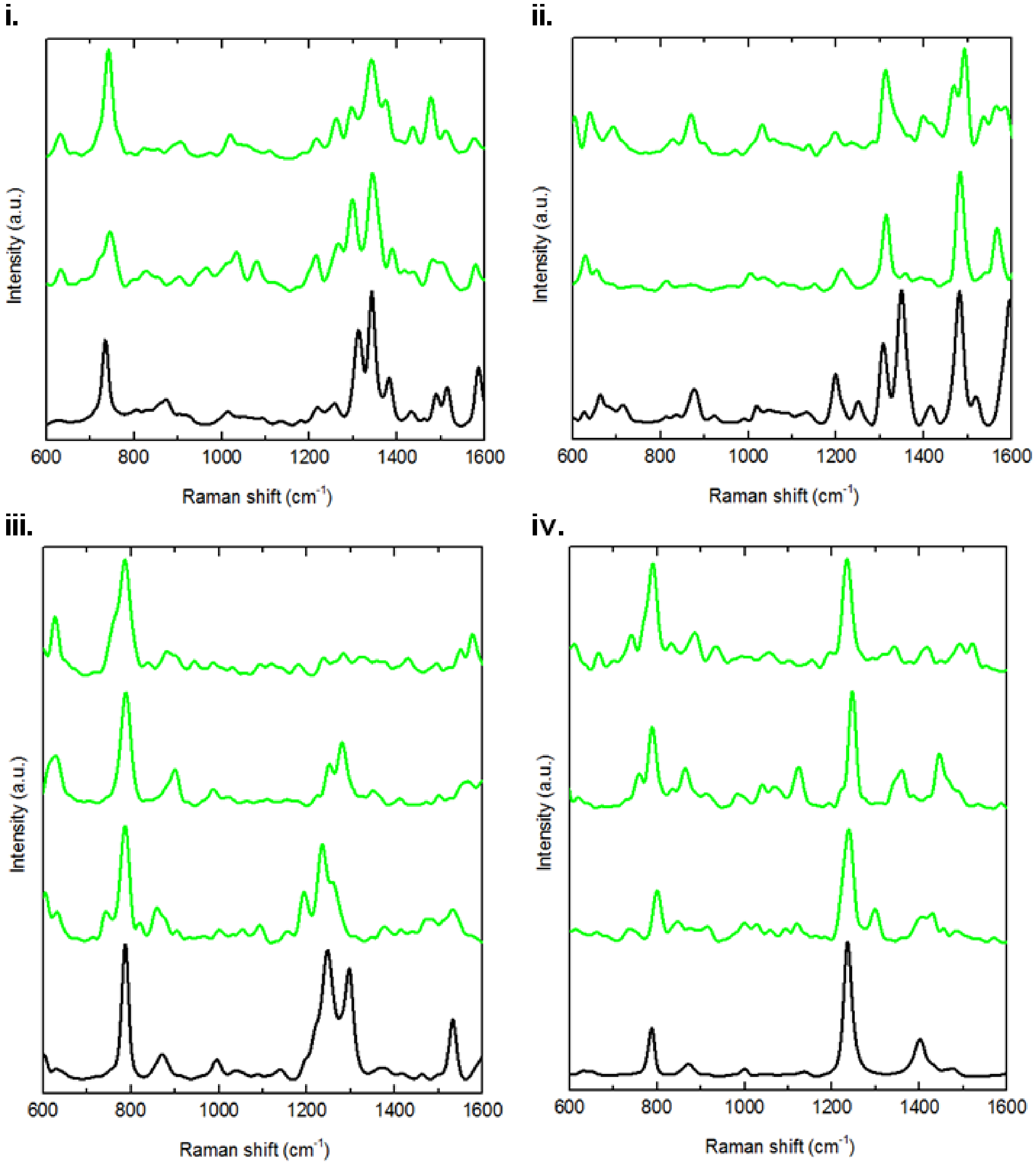

Supplementary Figure 7 | Raman spectra of i) adenosine, ii) guanosine, iii) cytidine, and iv) uridine determined experimentally (black) and theoretically predicted (green) for specific ribonucleoside conformer regions, based on sampling of local dynamics by AIMD. In order (from top to bottom) and in green are *syn*/south, *syn*/north, and *anti*/north-south conformer regions each show strong differences in relative peak intensities. Combining these spectra by optimizing the weighting of each to experiment leads to the prediction of solution conformer populations in Table 1.

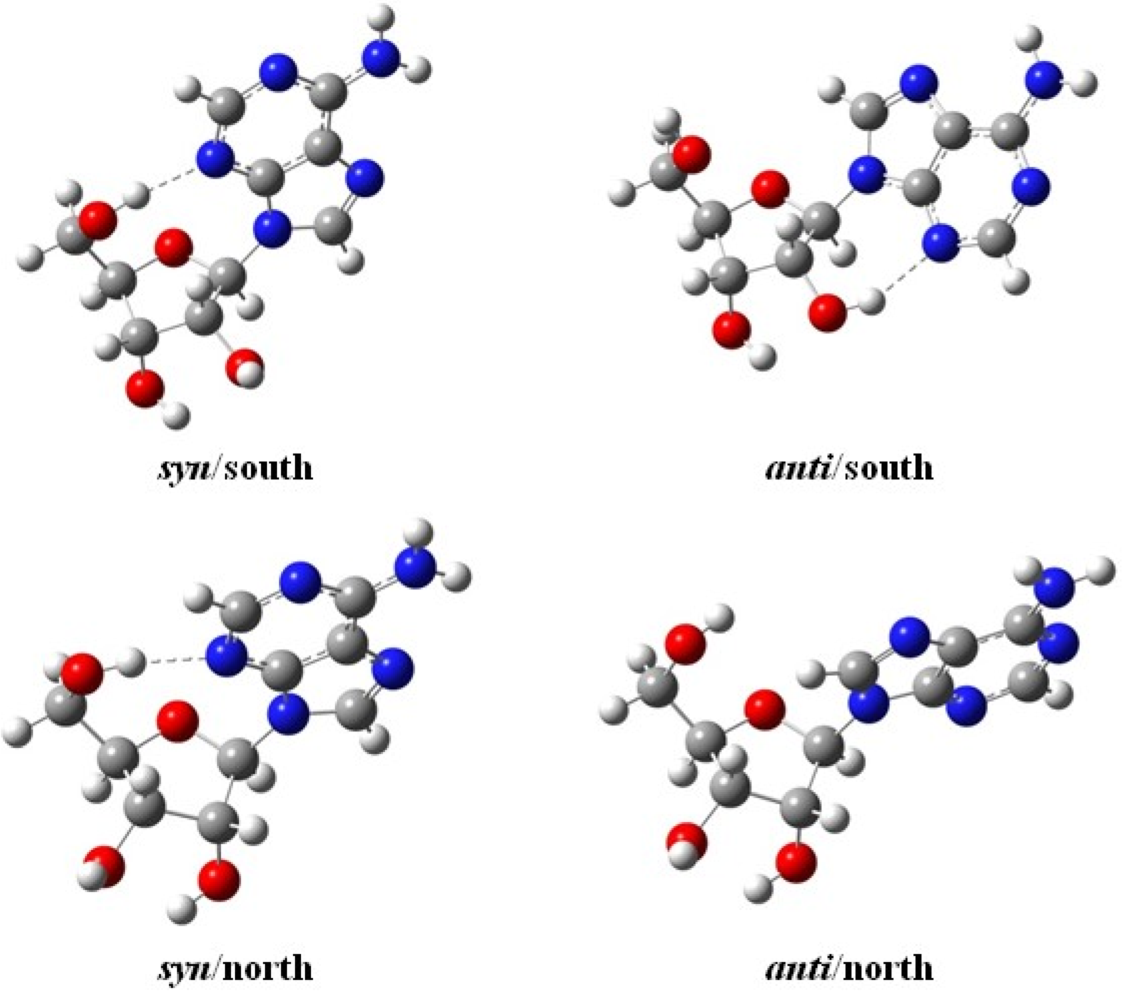

Supplementary Figure 8 | Optimized solvated conformers of adenosine, with water molecules removed for clarity. The observed *syn* preference for purine nucleosides in solution may be explained by the presence of a stabilizing intra-molecular hydrogen bonding interaction. The energies relative to the *syn*/south conformer are +9.0, +5.4, and +24.3 kJ mol^{-1} for *syn*/north, *anti*/south, and *anti*/north, respectively.

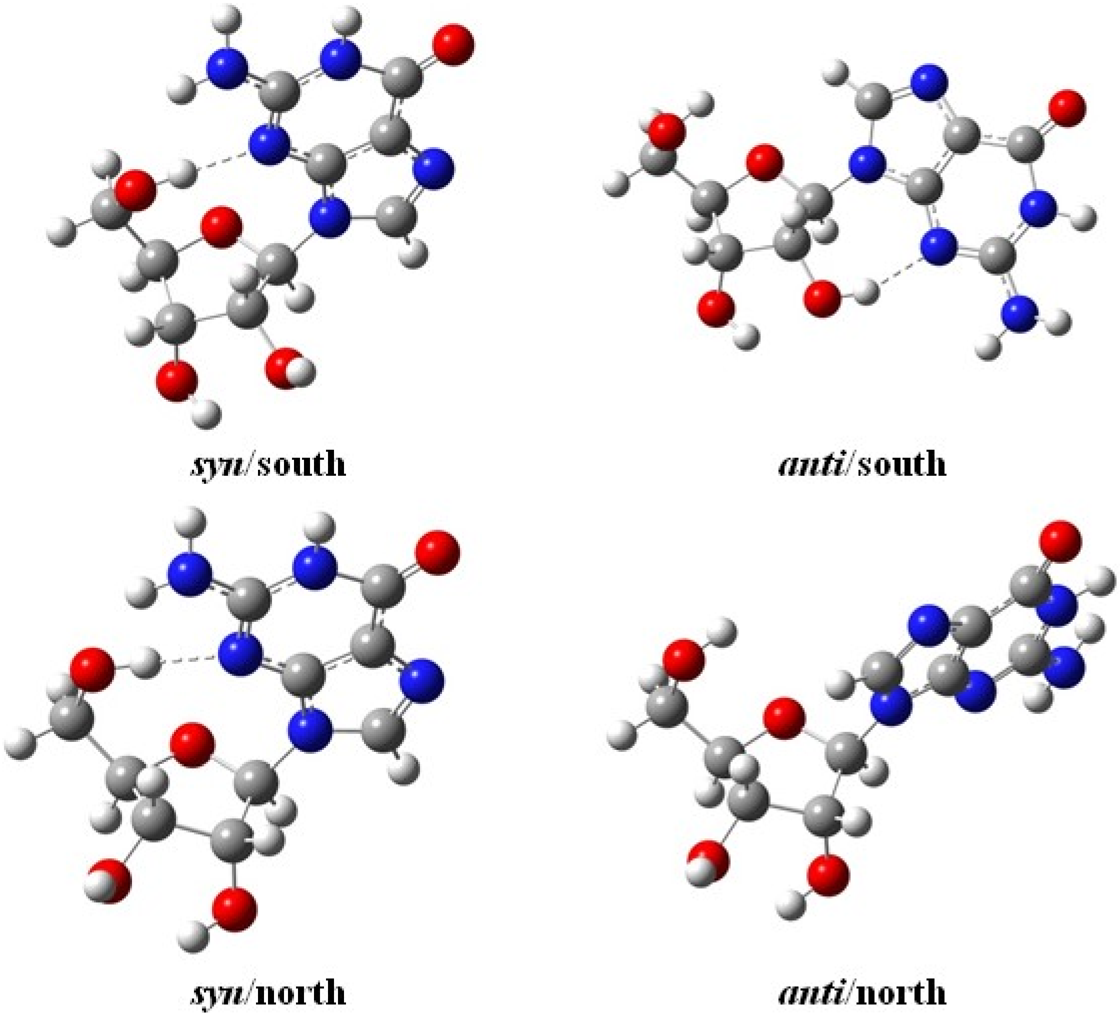

Supplementary Figure 9 | Optimized solvated conformers of guanosine, with water molecules removed for clarity. The observed *syn* preference for purine nucleosides in solution may be explained by the presence of a stabilizing intra-molecular hydrogen bonding interaction. The energies relative to the *syn*/south conformer are +6.2, +10.7, and +19.5 kJ mol^{-1} for *syn*/north, *anti*/south, and *anti*/north, respectively.

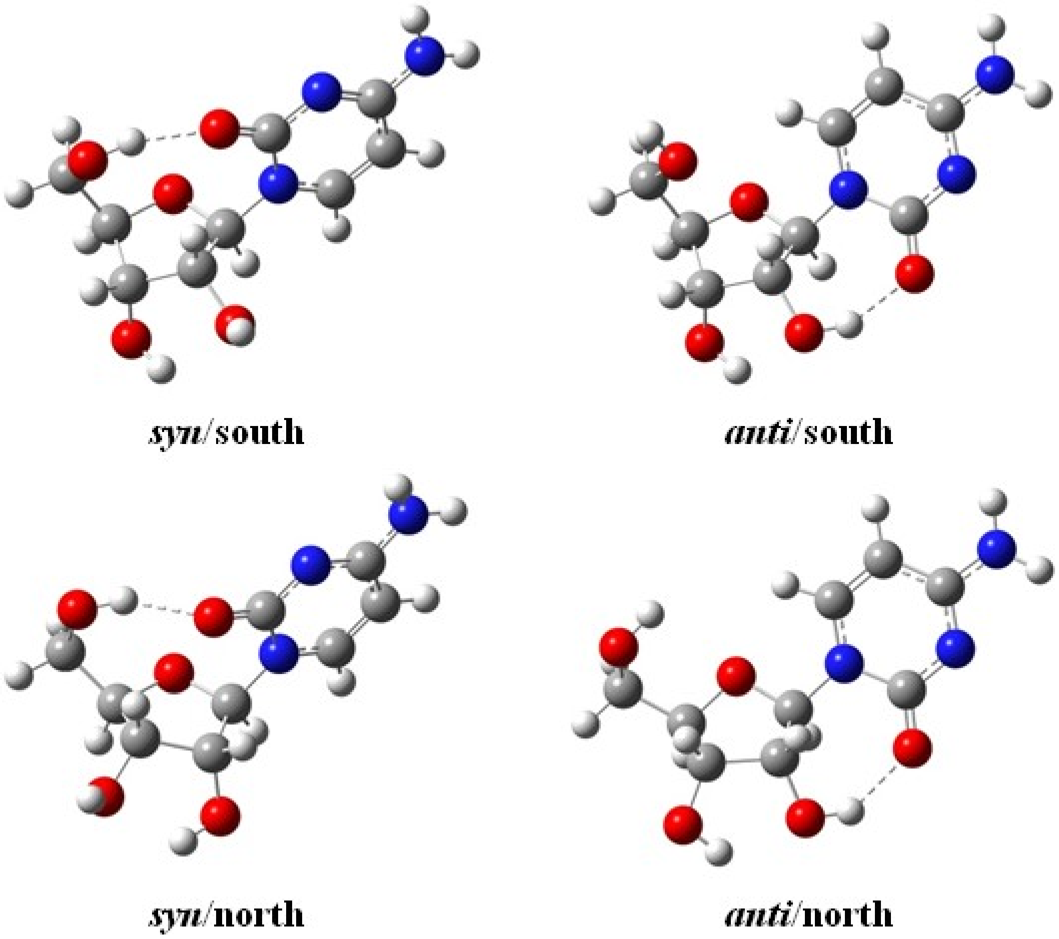

Supplementary Figure 10 | Optimized solvated conformers of cytidine, with water molecules removed for clarity. The observed *anti* preference for pyrimidine nucleosides in solution may be explained by the presence of a stabilizing intra-molecular hydrogen bonding interaction with the 2-OH group. The energies relative to the *syn*/south conformer are +2.1, -7.7, and -3.6 kJ mol^{-1} for *syn*/north, *anti*/south, and *anti*/north, respectively.

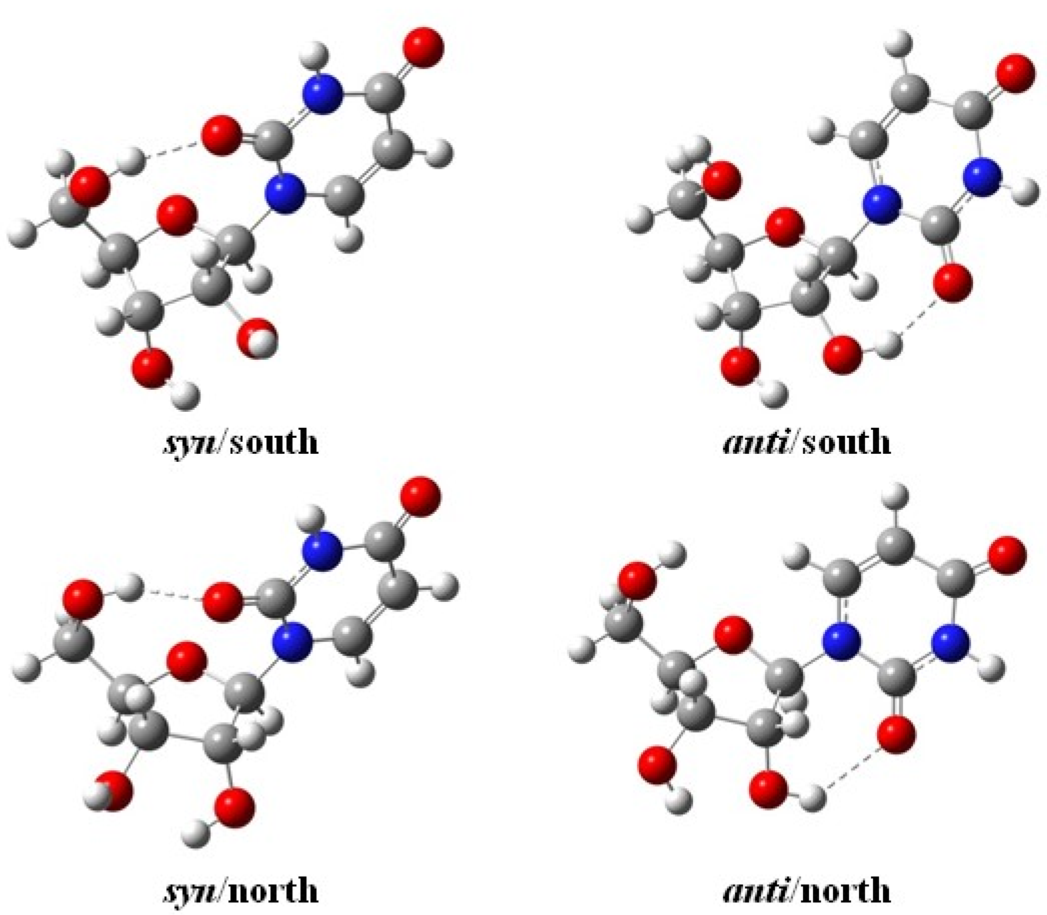

Supplementary Figure 11 | Optimized solvated conformers of uridine, with water molecules removed for clarity. The observed *anti* preference for pyrimidine nucleosides in solution may be explained by the presence of a stabilizing intra-molecular hydrogen bonding interaction with the 2-OH group. The energies relative to the *syn*/south conformer are +3.1, -4.7, and +3.2 kJ mol^{-1} for *syn*/north, *anti*/south, and *anti*/north, respectively.

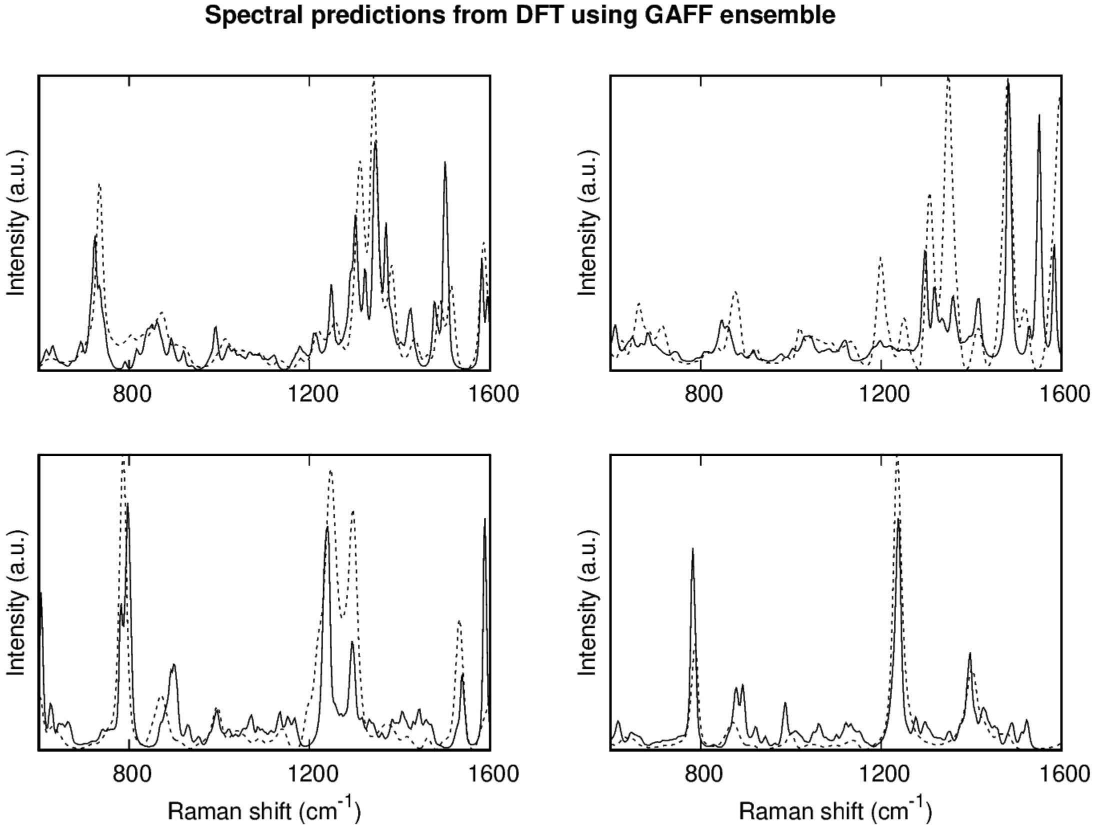

Base	χ^2 OPLS	χ^2 GAFF
**A**	3.7×10^{8}	2.6×10^{8}
**G**	2.3×10^{22}	2.1×10^{22}
**C**	1.2×10^{11}	1.3×10^{11}
**U**	8.7×10^{10}	8.8×10^{10}

Supplementary Figure 12 | Comparison of DFT calculations from the GAFF ensemble (1000 conformers from each MD simulation) with experiment. Top four panels: adenosine, top left, guanosine, top right, cytidine, bottom left, and uridine, bottom right. Solid lines are the theoretical predictions and the broken lines are the experimental measurements. Table at the bottom shows the χ^2 deviation between the experiment and theory for DFT predictions using OPLS and GAFF force fields (see Supplementary Table 3 for details of the force fields).

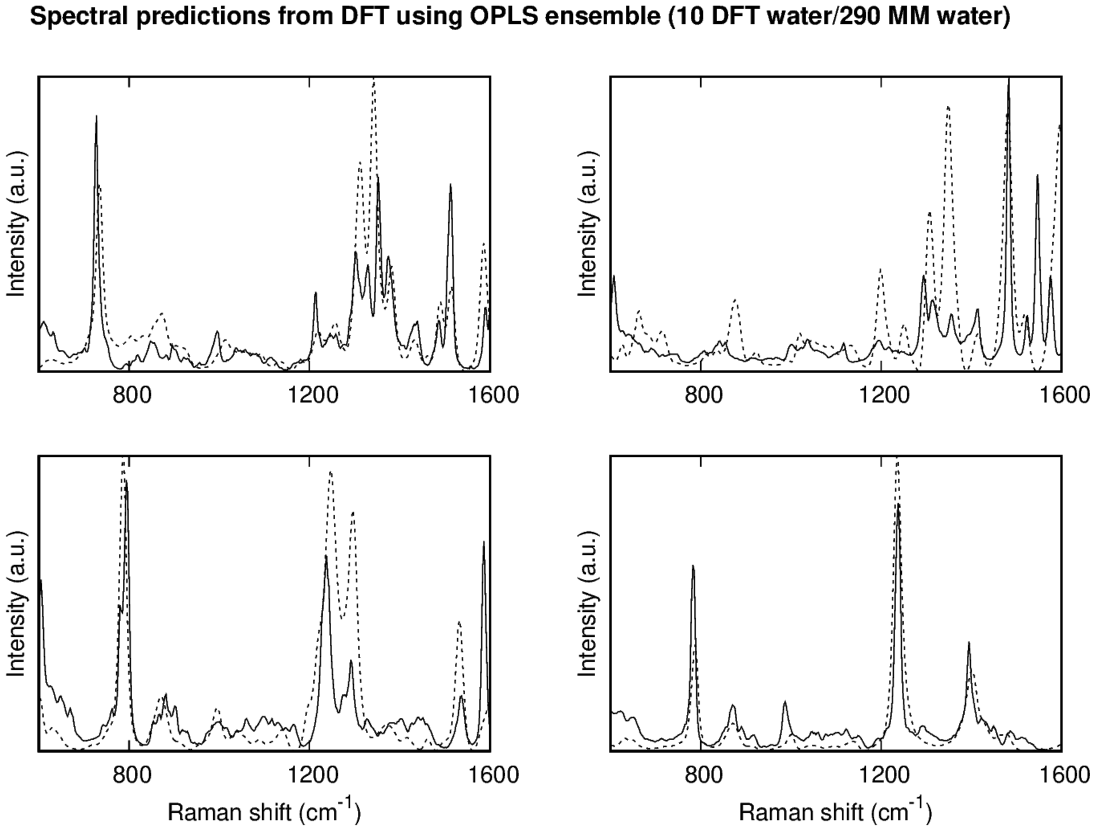

Base	χ^2 MM water	χ^2 DFT/MM water
**A**	3.7×10^{8}	3.2×10^{8}
**G**	2.3×10^{22}	2.3×10^{22}
**C**	1.2×10^{11}	1.2×10^{11}
**U**	8.7×10^{10}	8.5×10^{10}

Supplementary Figure 13 | Comparison of DFT calculations from the OPLS ensemble but with the 10 closest water molecules at the high-level B3LYP DFT approximation and 290 water molecules at the MM approximation (200 conformers from each MD simulation) with experiment. Top four panels: adenosine, top left, guanosine, top right, cytidine, bottom left, and uridine, bottom right. Solid lines are the theoretical predictions and the broken lines are the experimental measurements. Table at the bottom shows the χ^2 deviation between the experiment and theory for DFT predictions using the OPLS force fields with all MM water and 10 DFT/290 MM water.
