# Supplementary material for: Deconvolution of conformational exchange from Raman spectra of aqueous RNA nucleosides
Source: Commun Chem. 2020 May 6;3:56. doi: 10.1038/s42004-020-0298-x (PMC9814580; doi:10.1038/s42004-020-0298-x)
Supplement: Supplementary file 2 — Description of Additional Supplementary Files [file 42004_2020_298_MOESM2_ESM.pdf]

Description of Additional Supplementary Files

Deconvolution of Conformational Exchange from Raman Spectra of Aqueous RNA Nucleosides

SUPPLEMENTARY FILE 1

1. File Name: LineShape_program.cpp

Description. This is a C++ program allows fitting to the Raman experimental data with variable linewidth.

Compile the program as follows:

```
c++ LineShape_program.cpp -o LineShape_program
```

Run the program with an input file and output to a text file:

```
./LineShape_program example_input.txt > example_output.txt
```

example_input.txt below: first line is the path to the Raman spectrum,

which is a list of pairs (wavenumber, intensity) on successive lines

below are Guassian09 output files that have been run with the

freq=raman keyword (as many conformers as required).

```
Raw_data/adenosine_base_ave_23mar2018.txt
nuc_gau7/nuc_a_gau7/nuc_a_5/nuc_a_5.log
nuc_gau7/nuc_a_gau7/nuc_a_10/nuc_a_10.log
nuc_gau7/nuc_a_gau7/nuc_a_15/nuc_a_15.log
nuc_gau7/nuc_a_gau7/nuc_a_20/nuc_a_20.log
nuc_gau7/nuc_a_gau7/nuc_a_25/nuc_a_25.log
nuc_gau7/nuc_a_gau7/nuc_a_30/nuc_a_30.log
nuc_gau7/nuc_a_gau7/nuc_a_35/nuc_a_35.log
```

The output file will contain the optimised predicted spectrum.

SUPPLEMENTARY FILE 2

2. File Name: LineShape_program_fixed_line.cpp

Description: This is a derivative of Supplementary File 1 that allows fitting to the Raman experimental data with fixed 5cm-1 linewidth

(as used in Figure 3)

Compile the program as follows:

```
c++ LineShape_program_fixed_line.cpp -o LineShape_program_fixed_line
```

Run the program with an input file and output to a text file:

```
./LineShape_program_fixed_line example_input.txt > example_output.txt
```

example_input.txt below: first line is the path to the Raman spectrum,

which is a list of pairs (wavenumber, intensity) on successive lines

below are Guassian09 output files that have been run with the

freq=raman keyword (as many conformers as required).

```
Raw_data/adenosine_base_ave_23mar2018.txt
nuc_gau7/nuc_a_gau7/nuc_a_5/nuc_a_5.log
nuc_gau7/nuc_a_gau7/nuc_a_10/nuc_a_10.log
nuc_gau7/nuc_a_gau7/nuc_a_15/nuc_a_15.log
nuc_gau7/nuc_a_gau7/nuc_a_20/nuc_a_20.log
nuc_gau7/nuc_a_gau7/nuc_a_25/nuc_a_25.log
nuc_gau7/nuc_a_gau7/nuc_a_30/nuc_a_30.log
nuc_gau7/nuc_a_gau7/nuc_a_35/nuc_a_35.log
```

The output file will contain the optimised predicted spectrum.

3. File Name: AIMD_fit.py

Description: The program used to implement equations (1) and (2) in the methods section "Combining *ab initio* molecular dynamics derived spectra of individual conformers." This python script fits the spectra from a number of individual conformers to an experimental spectrum. See https://github.com/couteiral/RNA-Analysis for further information. The parameters are already set to the values described in the methods section.

Check that the required python libraries are available on your system and run it via:

python AIMD_fit.py
